# Supplementary material for: HIVprotI: an integrated web based platform for prediction and design of HIV proteins inhibitors
Source: J Cheminform. 2018 Mar 9;10:12. doi: 10.1186/s13321-018-0266-y (PMC5845081; doi:10.1186/s13321-018-0266-y)
Supplement: Supplementary file 2 — Additional file 2. Source code of HIVProtI web server. [file 13321_2018_266_MOESM2_ESM.zip › HIVprotI_Source-code/hivprotI/weka-3-6-6/doc/weka/gui/beans/xml/XMLBeans.html]

XMLBeans


---


|  |  |  |
| --- | --- | --- |
| **Overview** \| **Package** \| **Class** \| **Tree** \| **Deprecated** \| **Index** \| **Help** \| **Weka's home** | |  |
| **PREV CLASS**   NEXT CLASS | **FRAMES**    **NO FRAMES**     **All Classes** |  |
| SUMMARY: NESTED \| FIELD \| CONSTR \| METHOD | DETAIL: FIELD \| CONSTR \| METHOD |  |


---


## weka.gui.beans.xml Class XMLBeans

```
java.lang.Object
  weka.core.xml.XMLSerialization
      weka.core.xml.XMLBasicSerialization
          weka.gui.beans.xml.XMLBeans
```

**All Implemented Interfaces:**: RevisionHandler

---

``` public class XMLBeans extends XMLBasicSerialization ```

This class serializes and deserializes a KnowledgeFlow setup to and fro XML.
  

**Version:**
:   $Revision: 7411 $

**Author:**
:   FracPete (fracpete at waikato dot ac dot nz)

---

| **Field Summary** | |
| --- | --- |
| `static int` | `DATATYPE_LAYOUT`             the data that is about to be read/written contains a complete layout |
| `static int` | `DATATYPE_USERCOMPONENTS`             the data that is about to be read/written contains user-components, i.e., Metabeans |
| `static int` | `INDEX_BEANCONNECTIONS`             the index in the Vector, where the BeanConnections are stored (Instances and Connections are stored in a Vector and then serialized) |
| `static int` | `INDEX_BEANINSTANCES`             the index in the Vector, where the BeanInstances are stored (Instances and Connections are stored in a Vector and then serialized) |
| `static java.lang.String` | `VAL_ANIMATEDICONPATH`             the value of the animatedIconPath property |
| `static java.lang.String` | `VAL_ASSOCIATEDCONNECTIONS`             the value of the associatedConnections property |
| `static java.lang.String` | `VAL_BEAN`             the value of the bean property |
| `static java.lang.String` | `VAL_BEANCONTEXT`             the value of the beanContext property |
| `static java.lang.String` | `VAL_BLUE`             the value of the blue property |
| `static java.lang.String` | `VAL_COLOR`             the value of the color property |
| `static java.lang.String` | `VAL_CUSTOM_NAME`             the value of the customName property |
| `static java.lang.String` | `VAL_DIR`             the value of the dir property |
| `static java.lang.String` | `VAL_EVENTNAME`             the value of the eventname property |
| `static java.lang.String` | `VAL_FILE`             the value of the file property |
| `static java.lang.String` | `VAL_FONT`             the value of the font property |
| `static java.lang.String` | `VAL_GREEN`             the value of the green property |
| `static java.lang.String` | `VAL_HEIGHT`             the value of the height property |
| `static java.lang.String` | `VAL_HIDDEN`             the value of the hidden property |
| `static java.lang.String` | `VAL_ICONPATH`             the value of the iconpath property |
| `static java.lang.String` | `VAL_ID`             the value of the id property |
| `static java.lang.String` | `VAL_INPUTS`             the value of the input property |
| `static java.lang.String` | `VAL_INPUTSID`             the value of the input id property |
| `static java.lang.String` | `VAL_LOADER`             the value of the loader property |
| `static java.lang.String` | `VAL_LOCATION`             the value of the location property |
| `static java.lang.String` | `VAL_NAME`             the value of the value property |
| `static java.lang.String` | `VAL_OPTIONS`             the value of the options property |
| `static java.lang.String` | `VAL_ORIGINALCOORDS`             the value of the originalCoords property |
| `static java.lang.String` | `VAL_OUTPUTS`             the value of the outputs id property |
| `static java.lang.String` | `VAL_OUTPUTSID`             the value of the outputs property |
| `static java.lang.String` | `VAL_PREFIX`             the value of the prefix property |
| `static java.lang.String` | `VAL_RED`             the value of the red property |
| `static java.lang.String` | `VAL_RELATIONNAMEFORFILENAME`             the value of the relationNameForFilename property (Saver) |
| `static java.lang.String` | `VAL_RELATIVE_PATH` |
| `static java.lang.String` | `VAL_SAVER`             the value of the saver property |
| `static java.lang.String` | `VAL_SIZE`             the value of the size property |
| `static java.lang.String` | `VAL_SOURCEID`             the value of the source property |
| `static java.lang.String` | `VAL_STYLE`             the value of the style property |
| `static java.lang.String` | `VAL_SUBFLOW`             the value of the subFlow property |
| `static java.lang.String` | `VAL_TARGETID`             the value of the target property |
| `static java.lang.String` | `VAL_TEXT`             the value of the text property |
| `static java.lang.String` | `VAL_WIDTH`             the value of the width property |
| `static java.lang.String` | `VAL_X`             the value of the x property |
| `static java.lang.String` | `VAL_Y`             the value of the y property |

**Fields inherited from class weka.core.xml.XMLBasicSerialization**

`VAL_CELLS, VAL_KEY, VAL_MAPPING, VAL_VALUE`

**Fields inherited from class weka.core.xml.XMLSerialization**

`ATT_ARRAY, ATT_ARRAY_DEFAULT, ATT_CLASS, ATT_NAME, ATT_NULL, ATT_NULL_DEFAULT, ATT_PRIMITIVE, ATT_PRIMITIVE_DEFAULT, ATT_VERSION, DOCTYPE, ROOT_NODE, TAG_OBJECT, VAL_NO, VAL_ROOT, VAL_YES`


**Constructor Summary**

`XMLBeans(javax.swing.JComponent layout,
java.beans.beancontext.BeanContextSupport context)`
  
          initializes the serialization for layouts

`XMLBeans(javax.swing.JComponent layout,
java.beans.beancontext.BeanContextSupport context,
int datatype)`
  
          initializes the serialization for different types of data


| **Method Summary** | |
| --- | --- |
| `void` | `clear()`             generates internally a new XML document and clears also the IgnoreList and the mappings for the Read/Write-Methods |
| `int` | `getDataType()`             returns the type of data that is to be read/written |
| `java.lang.Object` | `readBeanConnection(org.w3c.dom.Element node)`             builds the BeanConnection from the given DOM node. |
| `java.lang.Object` | `readBeanInstance(org.w3c.dom.Element node)`             builds the BeanInstance from the given DOM node. |
| `java.lang.Object` | `readBeanVisual(org.w3c.dom.Element node)`             builds the BeanVisual from the given DOM node. |
| `java.lang.Object` | `readColor(org.w3c.dom.Element node)`             builds the Color from the given DOM node. |
| `java.lang.Object` | `readColorUIResource(org.w3c.dom.Element node)`             builds the ColorUIResource from the given DOM node. |
| `java.lang.Object` | `readDimension(org.w3c.dom.Element node)`             builds the Dimension from the given DOM node. |
| `java.lang.Object` | `readFont(org.w3c.dom.Element node)`             builds the Font from the given DOM node. |
| `java.lang.Object` | `readFontUIResource(org.w3c.dom.Element node)`             builds the FontUIResource from the given DOM node. |
| `java.lang.Object` | `readLoader(org.w3c.dom.Element node)`             builds the Loader from the given DOM node. |
| `java.lang.Object` | `readMetaBean(org.w3c.dom.Element node)`             builds the MetaBean from the given DOM node. |
| `java.lang.Object` | `readPoint(org.w3c.dom.Element node)`             builds the Point from the given DOM node. |
| `java.lang.Object` | `readSaver(org.w3c.dom.Element node)`             builds the Saver from the given DOM node. |
| `void` | `setDataType(int value)`             sets what kind of data is to be read/written |
| `org.w3c.dom.Element` | `writeBeanConnection(org.w3c.dom.Element parent, java.lang.Object o, java.lang.String name)`             adds the given BeanConncetion to a DOM structure. |
| `org.w3c.dom.Element` | `writeBeanInstance(org.w3c.dom.Element parent, java.lang.Object o, java.lang.String name)`             adds the given BeanInstance to a DOM structure. |
| `org.w3c.dom.Element` | `writeBeanLoader(org.w3c.dom.Element parent, java.lang.Object o, java.lang.String name)`             adds the given Loader (a bean) to a DOM structure. |
| `org.w3c.dom.Element` | `writeBeanSaver(org.w3c.dom.Element parent, java.lang.Object o, java.lang.String name)`             adds the given Saver (a bean) to a DOM structure. |
| `org.w3c.dom.Element` | `writeBeanVisual(org.w3c.dom.Element parent, java.lang.Object o, java.lang.String name)`             adds the given BeanVisual to a DOM structure. |
| `org.w3c.dom.Element` | `writeColor(org.w3c.dom.Element parent, java.lang.Object o, java.lang.String name)`             adds the given Color to a DOM structure. |
| `org.w3c.dom.Element` | `writeColorUIResource(org.w3c.dom.Element parent, java.lang.Object o, java.lang.String name)`             adds the given ColorUIResource to a DOM structure. |
| `org.w3c.dom.Element` | `writeDimension(org.w3c.dom.Element parent, java.lang.Object o, java.lang.String name)`             adds the given Dimension to a DOM structure. |
| `org.w3c.dom.Element` | `writeFont(org.w3c.dom.Element parent, java.lang.Object o, java.lang.String name)`             adds the given Font to a DOM structure. |
| `org.w3c.dom.Element` | `writeFontUIResource(org.w3c.dom.Element parent, java.lang.Object o, java.lang.String name)`             adds the given FontUIResource to a DOM structure. |
| `org.w3c.dom.Element` | `writeLoader(org.w3c.dom.Element parent, java.lang.Object o, java.lang.String name)`             adds the given Loader to a DOM structure. |
| `org.w3c.dom.Element` | `writeMetaBean(org.w3c.dom.Element parent, java.lang.Object o, java.lang.String name)`             adds the given MetaBean to a DOM structure. |
| `org.w3c.dom.Element` | `writePoint(org.w3c.dom.Element parent, java.lang.Object o, java.lang.String name)`             adds the given Point to a DOM structure. |
| `org.w3c.dom.Element` | `writeSaver(org.w3c.dom.Element parent, java.lang.Object o, java.lang.String name)`             adds the given Saver to a DOM structure. |

**Methods inherited from class weka.core.xml.XMLBasicSerialization**

`getRevision, readCollection, readCostMatrixOld, readDefaultListModel, readMap, readMatrix, readMatrixOld, writeCollection, writeCostMatrixOld, writeDefaultListModel, writeMap, writeMatrix, writeMatrixOld`

**Methods inherited from class weka.core.xml.XMLSerialization**

`fromXML, getVersion, main, read, read, read, read, readBooleanFromXML, readByteFromXML, readCharFromXML, readDoubleFromXML, readFloatFromXML, readFromXML, readFromXML, readIntFromXML, readLongFromXML, readShortFromXML, toXML, write, write, write, write, writeToXML`

**Methods inherited from class java.lang.Object**

`equals, getClass, hashCode, notify, notifyAll, toString, wait, wait, wait`

**Field Detail**

### VAL\_ID

```
public static final java.lang.String VAL_ID
```

:   the value of the id property

    **See Also:**: Constant Field Values

---


### VAL\_X

```
public static final java.lang.String VAL_X
```

:   the value of the x property

    **See Also:**: Constant Field Values

---


### VAL\_Y

```
public static final java.lang.String VAL_Y
```

:   the value of the y property

    **See Also:**: Constant Field Values

---


### VAL\_BEAN

```
public static final java.lang.String VAL_BEAN
```

:   the value of the bean property

    **See Also:**: Constant Field Values

---


### VAL\_CUSTOM\_NAME

```
public static final java.lang.String VAL_CUSTOM_NAME
```

:   the value of the customName property

    **See Also:**: Constant Field Values

---


### VAL\_SOURCEID

```
public static final java.lang.String VAL_SOURCEID
```

:   the value of the source property

    **See Also:**: Constant Field Values

---


### VAL\_TARGETID

```
public static final java.lang.String VAL_TARGETID
```

:   the value of the target property

    **See Also:**: Constant Field Values

---


### VAL\_EVENTNAME

```
public static final java.lang.String VAL_EVENTNAME
```

:   the value of the eventname property

    **See Also:**: Constant Field Values

---


### VAL\_HIDDEN

```
public static final java.lang.String VAL_HIDDEN
```

:   the value of the hidden property

    **See Also:**: Constant Field Values

---


### VAL\_FILE

```
public static final java.lang.String VAL_FILE
```

:   the value of the file property

    **See Also:**: Constant Field Values

---


### VAL\_DIR

```
public static final java.lang.String VAL_DIR
```

:   the value of the dir property

    **See Also:**: Constant Field Values

---


### VAL\_PREFIX

```
public static final java.lang.String VAL_PREFIX
```

:   the value of the prefix property

    **See Also:**: Constant Field Values

---


### VAL\_RELATIVE\_PATH

```
public static final java.lang.String VAL_RELATIVE_PATH
```

**See Also:**: Constant Field Values

---


### VAL\_OPTIONS

```
public static final java.lang.String VAL_OPTIONS
```

:   the value of the options property

    **See Also:**: Constant Field Values

---


### VAL\_SAVER

```
public static final java.lang.String VAL_SAVER
```

:   the value of the saver property

    **See Also:**: Constant Field Values

---


### VAL\_LOADER

```
public static final java.lang.String VAL_LOADER
```

:   the value of the loader property

    **See Also:**: Constant Field Values

---


### VAL\_TEXT

```
public static final java.lang.String VAL_TEXT
```

:   the value of the text property

    **See Also:**: Constant Field Values

---


### VAL\_BEANCONTEXT

```
public static final java.lang.String VAL_BEANCONTEXT
```

:   the value of the beanContext property

    **See Also:**: Constant Field Values

---


### VAL\_WIDTH

```
public static final java.lang.String VAL_WIDTH
```

:   the value of the width property

    **See Also:**: Constant Field Values

---


### VAL\_HEIGHT

```
public static final java.lang.String VAL_HEIGHT
```

:   the value of the height property

    **See Also:**: Constant Field Values

---


### VAL\_RED

```
public static final java.lang.String VAL_RED
```

:   the value of the red property

    **See Also:**: Constant Field Values

---


### VAL\_GREEN

```
public static final java.lang.String VAL_GREEN
```

:   the value of the green property

    **See Also:**: Constant Field Values

---


### VAL\_BLUE

```
public static final java.lang.String VAL_BLUE
```

:   the value of the blue property

    **See Also:**: Constant Field Values

---


### VAL\_NAME

```
public static final java.lang.String VAL_NAME
```

:   the value of the value property

    **See Also:**: Constant Field Values

---


### VAL\_STYLE

```
public static final java.lang.String VAL_STYLE
```

:   the value of the style property

    **See Also:**: Constant Field Values

---


### VAL\_LOCATION

```
public static final java.lang.String VAL_LOCATION
```

:   the value of the location property

    **See Also:**: Constant Field Values

---


### VAL\_SIZE

```
public static final java.lang.String VAL_SIZE
```

:   the value of the size property

    **See Also:**: Constant Field Values

---


### VAL\_COLOR

```
public static final java.lang.String VAL_COLOR
```

:   the value of the color property

    **See Also:**: Constant Field Values

---


### VAL\_FONT

```
public static final java.lang.String VAL_FONT
```

:   the value of the font property

    **See Also:**: Constant Field Values

---


### VAL\_ICONPATH

```
public static final java.lang.String VAL_ICONPATH
```

:   the value of the iconpath property

    **See Also:**: Constant Field Values

---


### VAL\_ANIMATEDICONPATH

```
public static final java.lang.String VAL_ANIMATEDICONPATH
```

:   the value of the animatedIconPath property

    **See Also:**: Constant Field Values

---


### VAL\_ASSOCIATEDCONNECTIONS

```
public static final java.lang.String VAL_ASSOCIATEDCONNECTIONS
```

:   the value of the associatedConnections property

    **See Also:**: Constant Field Values

---


### VAL\_INPUTS

```
public static final java.lang.String VAL_INPUTS
```

:   the value of the input property

    **See Also:**: Constant Field Values

---


### VAL\_INPUTSID

```
public static final java.lang.String VAL_INPUTSID
```

:   the value of the input id property

    **See Also:**: Constant Field Values

---


### VAL\_OUTPUTS

```
public static final java.lang.String VAL_OUTPUTS
```

:   the value of the outputs id property

    **See Also:**: Constant Field Values

---


### VAL\_OUTPUTSID

```
public static final java.lang.String VAL_OUTPUTSID
```

:   the value of the outputs property

    **See Also:**: Constant Field Values

---


### VAL\_SUBFLOW

```
public static final java.lang.String VAL_SUBFLOW
```

:   the value of the subFlow property

    **See Also:**: Constant Field Values

---


### VAL\_ORIGINALCOORDS

```
public static final java.lang.String VAL_ORIGINALCOORDS
```

:   the value of the originalCoords property

    **See Also:**: Constant Field Values

---


### VAL\_RELATIONNAMEFORFILENAME

```
public static final java.lang.String VAL_RELATIONNAMEFORFILENAME
```

:   the value of the relationNameForFilename property (Saver)

    **See Also:**: Constant Field Values

---


### INDEX\_BEANINSTANCES

```
public static final int INDEX_BEANINSTANCES
```

:   the index in the Vector, where the BeanInstances are stored
    (Instances and Connections are stored in a Vector and then serialized)

    **See Also:**: Constant Field Values

---


### INDEX\_BEANCONNECTIONS

```
public static final int INDEX_BEANCONNECTIONS
```

:   the index in the Vector, where the BeanConnections are stored
    (Instances and Connections are stored in a Vector and then serialized)

    **See Also:**: Constant Field Values

---


### DATATYPE\_LAYOUT

```
public static final int DATATYPE_LAYOUT
```

:   the data that is about to be read/written contains a complete layout

    **See Also:**: `m_DataType`, Constant Field Values

---


### DATATYPE\_USERCOMPONENTS

```
public static final int DATATYPE_USERCOMPONENTS
```

:   the data that is about to be read/written contains user-components, i.e.,
    Metabeans

    **See Also:**: `m_DataType`, Constant Field Values


**Constructor Detail**

### XMLBeans

```
public XMLBeans(javax.swing.JComponent layout,
                java.beans.beancontext.BeanContextSupport context)
         throws java.lang.Exception
```

:   initializes the serialization for layouts

    **Parameters:**: `layout` - the component that manages the layout: `context` - the bean context support to use **Throws:**: `java.lang.Exception` - if initialization fails

---


### XMLBeans

```
public XMLBeans(javax.swing.JComponent layout,
                java.beans.beancontext.BeanContextSupport context,
                int datatype)
         throws java.lang.Exception
```

:   initializes the serialization for different types of data

    **Parameters:**: `layout` - the component that manages the layout: `context` - the bean context support to use: `datatype` - the type of data to read/write **Throws:**: `java.lang.Exception` - if initialization fails


**Method Detail**

### setDataType

```
public void setDataType(int value)
```

:   sets what kind of data is to be read/written

    :   **Parameters:**: `value` - the type of data **See Also:**: `m_DataType`

---


### getDataType

```
public int getDataType()
```

:   returns the type of data that is to be read/written

    :   **Returns:**: the type of data **See Also:**: `m_DataType`

---


### clear

```
public void clear()
           throws java.lang.Exception
```

:   generates internally a new XML document and clears also the IgnoreList and
    the mappings for the Read/Write-Methods

    :   **Overrides:**: `clear` in class `XMLBasicSerialization`
    :   **Throws:**: `java.lang.Exception` - if something goes wrong

---


### writeColor

```
public org.w3c.dom.Element writeColor(org.w3c.dom.Element parent,
                                      java.lang.Object o,
                                      java.lang.String name)
                               throws java.lang.Exception
```

:   adds the given Color to a DOM structure.

    :   **Parameters:**: `parent` - the parent of this object, e.g. the class this object is a member of: `o` - the Object to describe in XML: `name` - the name of the object **Returns:**: the node that was created **Throws:**: `java.lang.Exception` - if the DOM creation fails

---


### readColor

```
public java.lang.Object readColor(org.w3c.dom.Element node)
                           throws java.lang.Exception
```

:   builds the Color from the given DOM node.

    :   **Parameters:**: `node` - the associated XML node **Returns:**: the instance created from the XML description **Throws:**: `java.lang.Exception` - if instantiation fails

---


### writeDimension

```
public org.w3c.dom.Element writeDimension(org.w3c.dom.Element parent,
                                          java.lang.Object o,
                                          java.lang.String name)
                                   throws java.lang.Exception
```

:   adds the given Dimension to a DOM structure.

    :   **Parameters:**: `parent` - the parent of this object, e.g. the class this object is a member of: `o` - the Object to describe in XML: `name` - the name of the object **Returns:**: the node that was created **Throws:**: `java.lang.Exception` - if the DOM creation fails

---


### readDimension

```
public java.lang.Object readDimension(org.w3c.dom.Element node)
                               throws java.lang.Exception
```

:   builds the Dimension from the given DOM node.

    :   **Parameters:**: `node` - the associated XML node **Returns:**: the instance created from the XML description **Throws:**: `java.lang.Exception` - if instantiation fails

---


### writeFont

```
public org.w3c.dom.Element writeFont(org.w3c.dom.Element parent,
                                     java.lang.Object o,
                                     java.lang.String name)
                              throws java.lang.Exception
```

:   adds the given Font to a DOM structure.

    :   **Parameters:**: `parent` - the parent of this object, e.g. the class this object is a member of: `o` - the Object to describe in XML: `name` - the name of the object **Returns:**: the node that was created **Throws:**: `java.lang.Exception` - if the DOM creation fails

---


### readFont

```
public java.lang.Object readFont(org.w3c.dom.Element node)
                          throws java.lang.Exception
```

:   builds the Font from the given DOM node.

    :   **Parameters:**: `node` - the associated XML node **Returns:**: the instance created from the XML description **Throws:**: `java.lang.Exception` - if instantiation fails

---


### writePoint

```
public org.w3c.dom.Element writePoint(org.w3c.dom.Element parent,
                                      java.lang.Object o,
                                      java.lang.String name)
                               throws java.lang.Exception
```

:   adds the given Point to a DOM structure.

    :   **Parameters:**: `parent` - the parent of this object, e.g. the class this object is a member of: `o` - the Object to describe in XML: `name` - the name of the object **Returns:**: the node that was created **Throws:**: `java.lang.Exception` - if the DOM creation fails

---


### readPoint

```
public java.lang.Object readPoint(org.w3c.dom.Element node)
                           throws java.lang.Exception
```

:   builds the Point from the given DOM node.

    :   **Parameters:**: `node` - the associated XML node **Returns:**: the instance created from the XML description **Throws:**: `java.lang.Exception` - if instantiation fails

---


### writeColorUIResource

```
public org.w3c.dom.Element writeColorUIResource(org.w3c.dom.Element parent,
                                                java.lang.Object o,
                                                java.lang.String name)
                                         throws java.lang.Exception
```

:   adds the given ColorUIResource to a DOM structure.

    :   **Parameters:**: `parent` - the parent of this object, e.g. the class this object is a member of: `o` - the Object to describe in XML: `name` - the name of the object **Returns:**: the node that was created **Throws:**: `java.lang.Exception` - if the DOM creation fails

---


### readColorUIResource

```
public java.lang.Object readColorUIResource(org.w3c.dom.Element node)
                                     throws java.lang.Exception
```

:   builds the ColorUIResource from the given DOM node.

    :   **Parameters:**: `node` - the associated XML node **Returns:**: the instance created from the XML description **Throws:**: `java.lang.Exception` - if instantiation fails

---


### writeFontUIResource

```
public org.w3c.dom.Element writeFontUIResource(org.w3c.dom.Element parent,
                                               java.lang.Object o,
                                               java.lang.String name)
                                        throws java.lang.Exception
```

:   adds the given FontUIResource to a DOM structure.

    :   **Parameters:**: `parent` - the parent of this object, e.g. the class this object is a member of: `o` - the Object to describe in XML: `name` - the name of the object **Returns:**: the node that was created **Throws:**: `java.lang.Exception` - if the DOM creation fails

---


### readFontUIResource

```
public java.lang.Object readFontUIResource(org.w3c.dom.Element node)
                                    throws java.lang.Exception
```

:   builds the FontUIResource from the given DOM node.

    :   **Parameters:**: `node` - the associated XML node **Returns:**: the instance created from the XML description **Throws:**: `java.lang.Exception` - if instantiation fails

---


### writeBeanInstance

```
public org.w3c.dom.Element writeBeanInstance(org.w3c.dom.Element parent,
                                             java.lang.Object o,
                                             java.lang.String name)
                                      throws java.lang.Exception
```

:   adds the given BeanInstance to a DOM structure.

    :   **Parameters:**: `parent` - the parent of this object, e.g. the class this object is a member of: `o` - the Object to describe in XML: `name` - the name of the object **Returns:**: the node that was created **Throws:**: `java.lang.Exception` - if the DOM creation fails

---


### readBeanInstance

```
public java.lang.Object readBeanInstance(org.w3c.dom.Element node)
                                  throws java.lang.Exception
```

:   builds the BeanInstance from the given DOM node.

    :   **Parameters:**: `node` - the associated XML node **Returns:**: the instance created from the XML description **Throws:**: `java.lang.Exception` - if instantiation fails

---


### writeBeanConnection

```
public org.w3c.dom.Element writeBeanConnection(org.w3c.dom.Element parent,
                                               java.lang.Object o,
                                               java.lang.String name)
                                        throws java.lang.Exception
```

:   adds the given BeanConncetion to a DOM structure.

    :   **Parameters:**: `parent` - the parent of this object, e.g. the class this object is a member of: `o` - the Object to describe in XML: `name` - the name of the object **Returns:**: the node that was created **Throws:**: `java.lang.Exception` - if the DOM creation fails

---


### readBeanConnection

```
public java.lang.Object readBeanConnection(org.w3c.dom.Element node)
                                    throws java.lang.Exception
```

:   builds the BeanConnection from the given DOM node.

    :   **Parameters:**: `node` - the associated XML node **Returns:**: the instance created from the XML description **Throws:**: `java.lang.Exception` - if instantiation fails

---


### writeBeanLoader

```
public org.w3c.dom.Element writeBeanLoader(org.w3c.dom.Element parent,
                                           java.lang.Object o,
                                           java.lang.String name)
                                    throws java.lang.Exception
```

:   adds the given Loader (a bean) to a DOM structure.

    :   **Parameters:**: `parent` - the parent of this object, e.g. the class this object is a member of: `o` - the Object to describe in XML: `name` - the name of the object **Returns:**: the node that was created **Throws:**: `java.lang.Exception` - if the DOM creation fails

---


### writeBeanSaver

```
public org.w3c.dom.Element writeBeanSaver(org.w3c.dom.Element parent,
                                          java.lang.Object o,
                                          java.lang.String name)
                                   throws java.lang.Exception
```

:   adds the given Saver (a bean) to a DOM structure.

    :   **Parameters:**: `parent` - the parent of this object, e.g. the class this object is a member of: `o` - the Object to describe in XML: `name` - the name of the object **Returns:**: the node that was created **Throws:**: `java.lang.Exception` - if the DOM creation fails

---


### writeLoader

```
public org.w3c.dom.Element writeLoader(org.w3c.dom.Element parent,
                                       java.lang.Object o,
                                       java.lang.String name)
                                throws java.lang.Exception
```

:   adds the given Loader to a DOM structure.

    :   **Parameters:**: `parent` - the parent of this object, e.g. the class this object is a member of: `o` - the Object to describe in XML: `name` - the name of the object **Returns:**: the node that was created **Throws:**: `java.lang.Exception` - if the DOM creation fails

---


### readLoader

```
public java.lang.Object readLoader(org.w3c.dom.Element node)
                            throws java.lang.Exception
```

:   builds the Loader from the given DOM node.

    :   **Parameters:**: `node` - the associated XML node **Returns:**: the instance created from the XML description **Throws:**: `java.lang.Exception` - if instantiation fails

---


### writeSaver

```
public org.w3c.dom.Element writeSaver(org.w3c.dom.Element parent,
                                      java.lang.Object o,
                                      java.lang.String name)
                               throws java.lang.Exception
```

:   adds the given Saver to a DOM structure.

    :   **Parameters:**: `parent` - the parent of this object, e.g. the class this object is a member of: `o` - the Object to describe in XML: `name` - the name of the object **Returns:**: the node that was created **Throws:**: `java.lang.Exception` - if the DOM creation fails

---


### readSaver

```
public java.lang.Object readSaver(org.w3c.dom.Element node)
                           throws java.lang.Exception
```

:   builds the Saver from the given DOM node.

    :   **Parameters:**: `node` - the associated XML node **Returns:**: the instance created from the XML description **Throws:**: `java.lang.Exception` - if instantiation fails

---


### writeBeanVisual

```
public org.w3c.dom.Element writeBeanVisual(org.w3c.dom.Element parent,
                                           java.lang.Object o,
                                           java.lang.String name)
                                    throws java.lang.Exception
```

:   adds the given BeanVisual to a DOM structure.

    :   **Parameters:**: `parent` - the parent of this object, e.g. the class this object is a member of: `o` - the Object to describe in XML: `name` - the name of the object **Returns:**: the node that was created **Throws:**: `java.lang.Exception` - if the DOM creation fails

---


### readBeanVisual

```
public java.lang.Object readBeanVisual(org.w3c.dom.Element node)
                                throws java.lang.Exception
```

:   builds the BeanVisual from the given DOM node.

    :   **Parameters:**: `node` - the associated XML node **Returns:**: the instance created from the XML description **Throws:**: `java.lang.Exception` - if instantiation fails

---


### writeMetaBean

```
public org.w3c.dom.Element writeMetaBean(org.w3c.dom.Element parent,
                                         java.lang.Object o,
                                         java.lang.String name)
                                  throws java.lang.Exception
```

:   adds the given MetaBean to a DOM structure.

    :   **Parameters:**: `parent` - the parent of this object, e.g. the class this object is a member of: `o` - the Object to describe in XML: `name` - the name of the object **Returns:**: the node that was created **Throws:**: `java.lang.Exception` - if the DOM creation fails

---


### readMetaBean

```
public java.lang.Object readMetaBean(org.w3c.dom.Element node)
                              throws java.lang.Exception
```

:   builds the MetaBean from the given DOM node.

    :   **Parameters:**: `node` - the associated XML node **Returns:**: the instance created from the XML description **Throws:**: `java.lang.Exception` - if instantiation fails


---


|  |  |  |
| --- | --- | --- |
| **Overview** \| **Package** \| **Class** \| **Tree** \| **Deprecated** \| **Index** \| **Help** \| **Weka's home** | |  |
| **PREV CLASS**   NEXT CLASS | **FRAMES**    **NO FRAMES**     **All Classes** |  |
| SUMMARY: NESTED \| FIELD \| CONSTR \| METHOD | DETAIL: FIELD \| CONSTR \| METHOD |  |


---
